# Anhuienoside C Ameliorates Collagen-Induced Arthritis through Inhibition of MAPK and NF-κB Signaling Pathways

**DOI:** 10.3389/fphar.2017.00299

**Published:** 2017-05-26

**Authors:** Qing Liu, Xu-Hui Xiao, Liu-Bing Hu, Hui-Yang Jie, Ying Wang, Wen-Cai Ye, Man-Mei Li, Zhong Liu

**Affiliations:** ^1^Guangdong Province Key Laboratory of Pharmacodynamic Constituents of TCM and New Drugs Research, Jinan UniversityGuangzhou, China; ^2^College of Pharmacy, Xiangnan UniversityChenzhou, China; ^3^Guangzhou Jinan Biomedicine Research and Development Center, Guangdong Provincial Key Laboratory of Bioengineering Medicine, College of Life Science and Technology, Jinan UniversityGuangzhou, China; ^4^College of Pharmacy, Jinan UniversityGuangzhou, China

**Keywords:** rheumatoid arthritis, *Anemone flaccida*, *Di Wu*, anhuienoside C, inflammatory cytokines

## Abstract

*Anemone flaccida* Fr. Schmidt (Ranunculaceae) (*Di Wu* in Chinese) is used to treat punch injuries and rheumatoid arthritis (RA). Our previous report has shown that crude triterpenoid saponins from *Anemone flaccida* exhibited anti-arthritic effects on type II collagen-induced arthritis in rats. Furthermore, anhuienoside C (AC), a saponin compound isolated from *A. flaccida*, was observed to suppress the nitric oxide production in lipopolysaccharide (LPS)-treated macrophage RAW 264.7 cells. In this study, we examined the effects of AC on the prevention and treatment of collagen-induced arthritis in a mouse model and evaluated the potential mechanisms involved. We observed that oral administration of AC significantly suppressed the paw swelling and arthritic score, decreased the body weight loss, and decreased the spleen index. Improvement in the disease severity was accompanied by the reduction of cluster of differentiation 68 (CD68)-positive cells in the ankle joint and inhibition of the pro-inflammatory cytokine tumor necrosis factor alpha (TNF-α) in the synovium of the joint. Mechanistic studies indicated that AC exerted its anti-inflammatory activity by inhibiting the mRNA expression levels of inducible nitric oxide synthase, cyclooxygenase-2, TNF-α, interleukin (IL)-1β, and IL-6 and by suppressing the production of inflammatory cytokines such as TNF-α, IL-1β, and IL-6 in LPS-treated RAW 264.7 cells. AC also blocked the LPS-induced activation of the extracellular signal-regulated kinase, c-Jun N-terminal kinase, and p38 mitogen-activated protein kinase pathways. Additionally, the LPS-induced activation of nuclear factor kappa-B (NF-κB) was significantly suppressed by AC treatment, as indicated by down-regulation of TLR4 and inhibition of the nuclear translocation of NF-κB p65 and by activation and degradation of the inhibitor of kappa B. These findings indicated that AC has a great potential to be developed as a therapeutic agent for human RA.

## Introduction

Rheumatoid arthritis is an autoimmune disease marked by chronic inflammation and synovial proliferation, leading to the cartilage damage and joint destruction (Vincent et al., 2012). Although the exact etiology and pathogenesis of RA are unclear, the disease is characterized, in particular, by the involvement of numerous activated macrophages and FLSs in the inflammation of the synovial membrane and cartilage-pannus junction (Garcia-Carbonell et al., 2016; Tang et al., 2016). These dysregulated immune cell interactions can induce the overproduction of proinflammatory cytokines such as IL-1β, IL-6, and TNF-α. It is reported that pro-inflammatory cytokines are activated by multiple signaling pathways, such as the MAPK and NF-κB pathways. In addition, exposure to pathogenic antigens such as LPS could induce TLR4 signaling pathway which subsequently induces NF-κB activation and expression of pro-inflammatory cytokines such as TNF-α, and IL-1β, IL-6, resulting in an imbalance between pro-inflammatory and anti-inflammatory cytokine activity and contributes to the pathogenesis of RA (Fu et al., 2013; Zhu et al., 2016).

Nonsteroidal anti-inflammatory drugs are widely approved for the alleviation of pain as well as inflammatory and autoimmune components of the disease, without reducing the cartilage and bone destruction of joints. Unfortunately, NSAIDs may cause various side effects or toxicity, such as gastrointestinal disorders and cardiovascular risk (Desai et al., 2014). Biologics, including TNF-α inhibitors (infliximab and adalimumab), IL-1β inhibitors (anakinra), and IL-6 inhibitors (tocilizumab and atlizumab), represent a prominent group of drugs used in the treatment of RA, but their administration may cause side effects and can interfere with the immune defense responses. Furthermore, their high cost makes the access to these drugs prohibitive for the general public (Bevaart et al., 2010; Li et al., 2013). Thus, there is an urgent need to develop novel therapeutic agents.

Natural products, in addition to their traditional uses, have served as important sources of bioactive compounds that have played significant roles in drug discovery and development processes. Dry rhizomes of *Anemone flaccida* Fr. Schmidt (Ranunculaceae), commonly known as *Di Wu* in China, are widely used as a Chinese folk medicine for fractures and to strengthen bones. Previous studies have demonstrated that triterpenoid saponins are the main chemicals and major bioactive constituents of this plant (Zhang et al., 2008; Han and Huang, 2009; Han et al., 2013). We have previously reported that crude triterpenoid saponins from *A. flaccida* (*Di Wu*) showed anti-arthritic effects on type II CIA in rats. Furthermore, AC, a saponin compound isolated from *A. flaccida*, was observed to suppress the NO production in LPS-treated macrophage RAW 264.7 cells (Huang et al., 2014; Liu et al., 2015). Therefore, in the present study, we examined the anti-rheumatic effects of AC in a mouse model and evaluated the potential mechanisms involved.

## Materials and Methods

### Reagents

**FIGURE 1:**
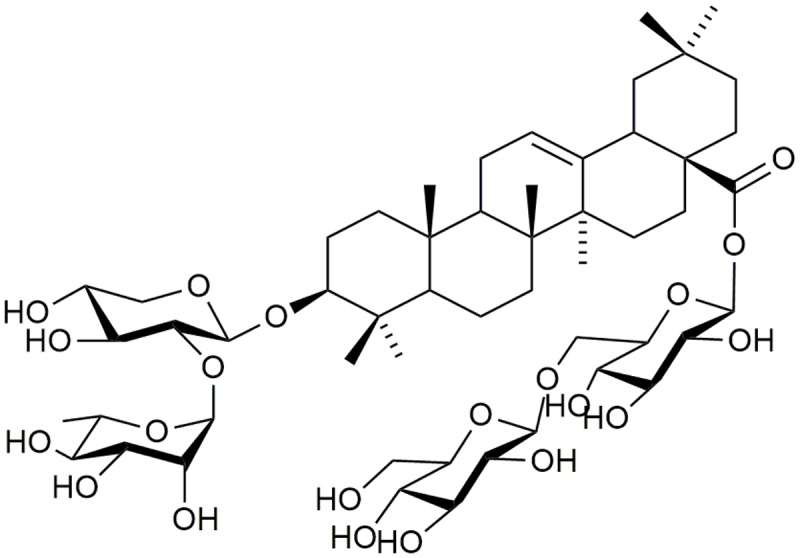
**Chemical structure of AC isolated from *Anemone flaccida***.

Anhuienoside C (purity > 97%; **Figure 1**) was isolated from dry rhizomes of *A. flaccida*, and the chemical structure was identified using ^1^H–^1^H correlation spectroscopy, heteronuclear single quantum coherence spectroscopy, heteronuclear multiple bond coherence, rotating-frame Overhauser spectroscopy, and ^1^H and ^13^C NMR analysis in our laboratory, as previously described (Huang et al., 2014). Dexamethasone used in mice was purchased from Guangdong Huanan Pharmaceutical Group Co., Ltd. (Guangzhou, China) and the dexamethasone was obtained from Aladdin (Shanghai, China). AC and dexamethasone were dissolved in distilled water. FBS, DMEM, and all other cell culture products were purchased from Life Technologies (Grand Island, NY, United States). Bovine CII, CFA (4 mg/mL), IFA (4 mg/mL), LPS (*Escherichia coli* 055:B5), and MTT were obtained from Sigma–Aldrich (St. Louis, MO, United States). LDH cytotoxicity detection kit was purchased from Jiancheng (Nanjing, China). TNF-α, IL-1β, and IL-6 ELISA kits were purchased from eBioscience (San Diego, CA, United States). Antibodies against inducible iNOS, phosphorylated (p)-p38, p38, p-ERK, ERK, p-c-JNK, JNK, p-IκBα, IκBα, NF-κB p65, and GAPDH were obtained from Cell Signaling Technology, Inc. (Beverly, MA, United States), Antibody against TLR4 was obtained from GeneTex (Irvine, CA, United States). A Pierce^TM^ BCA protein assay kit was purchased from Thermo Fisher Scientific, Inc. (Rockford, IL, United States). The murine macrophage cell line RAW 264.7 (ATCC TIB-7^TM^) was purchased from American Type Culture Collection (Manassas, VA, United States).

### Animals

Male DBA/1J mice (6- to 8-week-old), SPF grade, certified [No. SCXK (Shanghai) 2012-0002] were purchased from Shanghai SLAC Laboratory Animal Co., Ltd. (Shanghai, China). All mice were allowed to acclimate for 1 week before the experiments were started. The animals were maintained under SPF conditions at approximately 23 ± 1°C with a 12-h light/dark cycle and were given a standard diet and water *ad libitum*. All experimental procedures complied with the Guide for the Care and Use of Laboratory Animals and were approved by the local Animal Ethics Committee of Jinan University.

### Induction of Arthritis and Drug Administration

Experimental arthritis was induced in mice as described previously (Wen et al., 2012; Li et al., 2013). Briefly, 60 animals were randomly divided into the following six groups (*n* = 10 each): normal control; CIA; CIA + AC (20 mg/kg/day); CIA + AC (30 mg/kg/day); CIA + AC (40 mg/kg/day), and CIA + dexamethasone (0.2 mg/kg/day). Bovine CII was dissolved in 0.05 M acetic acid (2.0 mg/mL) and completely emulsified with CFA at a ratio of 1:1. The mice were immunized by intradermal injection of 100 μg of CII in CFA into the base of the tail. The day of the first immunization was defined as day 1. The second injection was administered on day 21 with an equal amount of CII emulsified in IFA. The CII solution and emulsions with CFA and IFA were always freshly prepared. On day 28 after the initial immunization, we used LPS (50 μg/mouse) to boost the arthritis incidence and severity, as previously reported (You et al., 2006; Wen et al., 2012). The mice in the normal control group did not receive the injection. To determine the effect of AC treatment on the onset of CIA, mice with a score of 2–4 were randomly divided into five equal cohorts 1 day after the LPS treatment, and AC administration was started. The mice in the AC treatment groups received an intragastric dose of AC (20, 30, or 40 mg/kg/day) for 21 days, while the positive control group received dexamethasone (0.2 mg/kg/day). The CIA and normal control groups were administered an equal volume of saline. Following the LPS challenge, the degree of arthritis was examined every 3 days. Meanwhile, the body weight of the mice was measured every 3 days, and changes in body weight were monitored. Arthritis symptoms were graded using a scoring system as previously described, with the maximum clinical score of 16 per mouse (Brand et al., 2007). The severity of arthritis was expressed as the mean arthritic index on a 0–4 scale according to the following criteria: 0, no edema or swelling; 1, swelling and erythema of one digit; 2, swelling and erythema of more than two digits or that limited to the foot; 3, slight edema and erythema from the ankle to the tarsal bone; and 4, severe edema and erythema involving the entire hind paw or forepaw. Thereafter, the mice were closely monitored and scored on alternate days in a blinded manner for signs of arthritis severity.

### Spleen Index Assay

The spleens were promptly removed and weighed when the animals were sacrificed on day 51 after the primary immunization. The spleen index was expressed as a ratio calculated as follows: ratio (mg/g) = spleen weight (mg)/body weight (g) × 10^3^.

### Histological Analysis of Paws

On day 51, all mice were sacrificed. The forepaws and hind paws were surgically removed and skinned. The right hind paws were fixed in 4% buffered formaldehyde, then decalcified in 12% disodium ethylenediaminetetraacetic acid for 1 month, dehydrated, and embedded in paraffin. Sections were cut along the longitudinal axis, mounted, and stained with H&E.

### Immunohistochemical Staining of Joint Tissues

For the quantitative analysis of macrophage infiltration and local TNF-α accumulation in joint tissue, commercially available monoclonal TNF-α and cluster of CD68 antibodies were used. Three slices were individually taken from each mouse, and four vision fields were randomly observed at a high magnification (×100).

### Cell Culture

RAW 264.7 cells were maintained in DMEM supplemented with 100 U/mL penicillin, 100 μg/mL streptomycin, 10% heat-inactivated FBS, 2 mM glutamine, 1 mM sodium pyruvate, and 4.5 g/L glucose at 37°C in a humidified atmosphere containing 5% CO_2_.

### LDH and MTT Assay

To determine the cytotoxicity of AC and dexamethasone, RAW 264.7 cells (5 × 10^3^ cells/well) were seeded in a 96-well plate containing DMEM supplemented with 10% FBS and incubated for 24 h until the cells were nearly confluent. AC and dexamethasone was added at different concentrations (10, 20, 40, and 80 μM) with or without LPS (100 ng/mL) to the cell culture medium, and the plate was incubated for an additional 24 h. The cells were centrifuged at 12,000 rpm for 4 min at room temperature. Then culture medium was carefully removed from each well in order to determine LDH activity by using an LDH cytotoxicity detection kit according to the protocol of the manufacturer. The absorbance of each well was measured at 490 nm using a microplate reader (Synergy HT; BioTek, United States). The relative LDH release is defined by the ratio of LDH released over total LDH in untreated cells. For the analysis of cell viability, cells was treated with the indicated various concentrations (10, 20, 40, and 80 μM) of AC or dexamethasone with or without LPS (100 ng/mL) for 24 h. The cells were washed twice with PBS and incubated with 30 μL of MTT (5 mg/mL) for 4 h at 37°C. The supernatant was discarded, and 100 μL of DMSO was added. After 15 min of incubation, absorbance was measured at 570 nm in a microplate reader (Synergy HT; BioTek, United States).

### Quantitative Real-Time Polymerase Chain Reaction Assay

Quantitative real-time polymerase chain reaction (PCR) was performed to determine whether AC regulated the mRNA expression of iNOS, COX-2, TNF-α, IL-1β, and IL-6 in RAW 264.7 cells following exposure to LPS (100 ng/mL) for 3 h. RAW 264.7 murine macrophages were pretreated with AC at different concentrations for 2 h before stimulation with 100 ng/mL LPS. After the incubation for an additional 3 h, total RNA was extracted using the TRIzol reagent (Invitrogen, Carlsbad, CA, United States), then quantified, and reverse-transcribed to cDNA. Relative quantitation of expression of the selected genes was performed in a LightCycler 480 system (Roche, Pleasanton, CA, United States) using a SYBR Green PCR master mix reagent kit (Takara, Dalian, China) and the PCR primers shown in **Table 1**. The cycling conditions were 95°C for 30 s, followed by 40 cycles of 95°C for 10 s, 57°C for 10 s, and 72°C for 10 s. A dissociation curve was generated in a cycle of 95°C for 5 s, 67°C for 1 min, and 97°C for 15 s. The mRNA expression levels were determined relative to the blank control after normalization to the GAPDH level using the 2^-ΔΔC_t_^ method. Analysis was carried out in triplicates. Control cells were grown under identical conditions without AC and LPS. In addition, RAW 264.7 cells were incubated under identical conditions in the absence of LPS with AC (40 μM) alone.

**Table 1:** Primers Used for Real-Time PCR.

Gene name		Primer sequence (5′-3′)	Product length (bp)
IL-1β	Forward	GAAATGCCACCTTTTGACAGTG	116
	Reverse	TGGATGCTCTCATCAGGACAG	
IL-6	Forward	TAGTCCTTCCTACCCCAATTTCC	76
	Reverse	TTGGTCCTTAGCCACTCCTTC	
COX-2	Forward	TTCAACACACTCTATCACTGGC	271
	Reverse	AGAAGCGTTTGCGGTACTCAT	
iNOS	Forward	GGAGTGACGGCAAACATGACT	127
	Reverse	TCGATGCACAACTGGGTGAAC	
TNF-α	Forward	GGGCCACCACGCTCTTC	104
	Reverse	GGTCTGGGCCATAGAACTGATG	
GAPDH	Forward	AGGTCGGTGTGAACGGATTTG	123
	Reverse	TGTAGACCATGTAGTTGAGGTCA	


### Measurement of TNF-α, IL-1β, and IL-6 levels in RAW 264.7 Cells

The effect of AC on the cytokine (TNF-α, IL-1β, and IL-6) release from RAW 264.7 cells was evaluated using ELISA assays. Briefly, RAW 264.7 murine macrophages (5 × 10^5^ cells/well) were cultured in a 6-well microplate for 24 h. The cells were pretreated with varying concentrations of AC for 1 h before stimulation with 100 ng/mL LPS. The activated cells were incubated for another 6 h to measure the TNF-α, IL-1β, and IL-6 secretion. The supernatants were collected, and the concentrations of TNF-α, IL-1β, and IL-6 were determined using specific ELISA kits according to the manufacturer’s protocols.

### Western Blot Analysis

RAW 264.7 cells (1.6 × 10^6^ cells/well) were seeded in a 100-mm dish for 24 h and then pretreated with varying concentrations of AC (10–40.0 μM) for 2 h, followed by LPS stimulation for 30 min. The cells were washed twice with ice-cold PBS, collected, lysed, and then centrifuged at 14,000 × *g* for 15 min at 4°C. The supernatant was collected, and protein concentrations were determined by the BCA protein assay. Total protein samples (20 μg) were separated by 8% (w/v) SDS-PAGE and transferred onto a PVDF membrane (Millipore, Billerica, MA, United States). All membranes were incubated for 1 h at room temperature with 5% (w/v) skim milk in Tris-buffered saline with Tween 20 (TBST) to block non-specific binding and then incubated overnight at 4°C with specific rabbit polyclonal antibodies (1:1,000) recognizing iNOS, p-ERK, ERK, p-JNK, JNK, p-p38, p38, p-IκBα, IκBα, and TLR4. The blots were rinsed three times with TBST buffer for 5 min and subsequently incubated with a goat anti-rabbit IgG horseradish peroxidase-conjugated secondary antibody for 2 h at room temperature. The signals were detected using an enhanced ECL system (Bio-Rad, Hercules, CA, United States) according to the manufacturer’s instructions.

### Detection of NF-κB Nuclear Translocation by Immunofluorescent Staining

RAW 264.7 cells (2 × 10^4^ cells/mL) were cultured in 15-mm culture dishes for 24 h and pretreated with AC for 2 h prior to treatment with 100 ng/mL LPS for 30 min. The cells were fixed, permeabilized, and immunostained with the anti-NF-κB p65 antibody and then with the secondary anti-goat antibody. Nuclear staining was visualized with DAPI (Sigma, St. Louis, MO, United States). Fluorescence signals were recorded using a confocal laser scan microscope LSM 510 (Zeiss, Germany).

### Statistical Analysis

Data are reported as the mean ± SD from three or more experiments using the GraphPad Prism 5.0 statistical package. The Student’s *t*-test and one-way analysis of variance were used for parametric analysis to compare groups and perform multi-group comparisons. The Mann–Whitney *U*-test was used to analyze non-parametric data. A *P*-value of less than 0.05 was considered to be statistically significant, and a *P*-value of less than 0.01 was considered very significant.

## Results

### Effects of AC on RA Symptoms and Body Weight in CIA Mice

**FIGURE 2:**
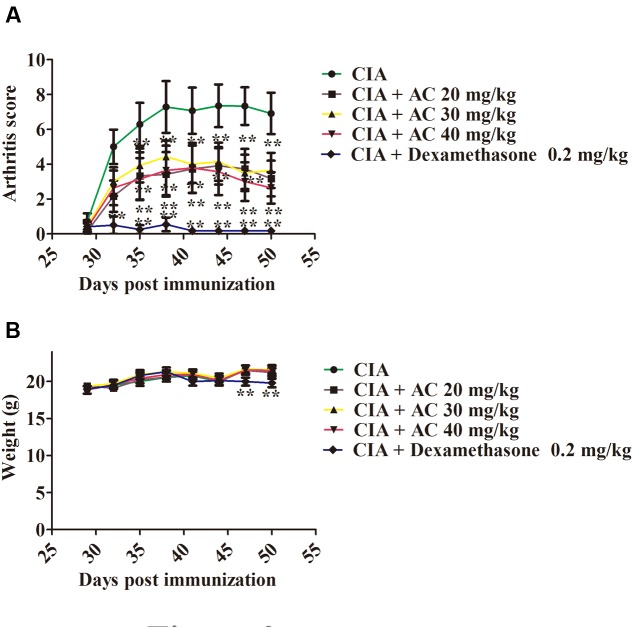
**The effect of AC on CIA mice.** **(A)** AC treatment ameliorates the paw edema volume and redness compared with those in the CIA mice. AC was administered intragastrically 1 day after LPS treatment (day 29 of the initial immunization) and every days thereafter until day 50. Mice receiving dexamethasone (0.2 mg/kg) were used as a positive control group. The clinical score was used to evaluate the progression of arthritis development. *^∗^P* < 0.05, ^∗∗^*P* < 0.01 for the treatment groups vs. the CIA group. **(B)** Body weight changes in mice. The data are expressed as the mean ± SD (*n* = 10). ^∗∗^*P* < 0.01 for the dexamethasone treatment group vs. the CIA group.

The anti-arthritic effects of AC on the progression of LPS-enhanced arthritis development were assessed in mice with CIA. The CIA mice developed severe swelling, redness, erythema, and joint rigidity of the forepaws and hind paws. In contrast, administration of AC (20, 30, and 40 mg/kg/day) significantly blocked the progression of arthritis development, and according to the mean arthritis scores, the severity of CIA was significantly attenuated by AC (**Figure 2A**).

Along with the development of arthritis, the relationship between the extent of paw swelling and the weight loss was investigated. As shown in **Figure 2B**, the AC treatment enhanced the body weight of the CIA mice, followed by a normal weight gain in the subsequent weeks, whereas the dexamethasone-treated mice showed a significant weight loss.

### Effects of AC on Spleen Index

**FIGURE 3:**
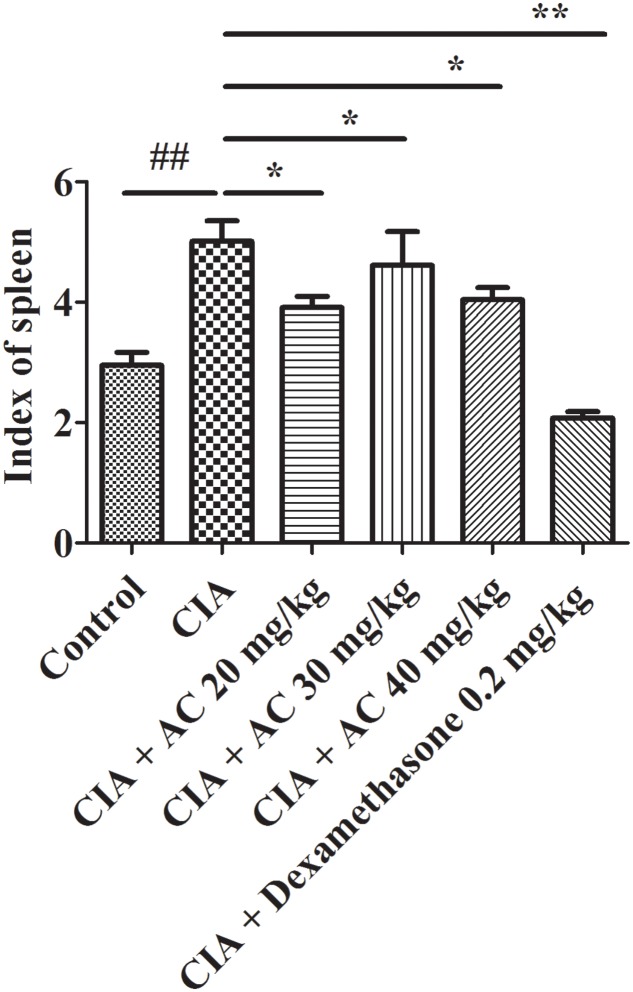
**Effects of AC on the spleen index of CIA mice.** The data represent the mean ± SD (*n* = 10). ^##^*P* < 0.01 compared with the normal control mice; ^∗^*P* < 0.05; ^∗∗^*P* < 0.01 compared with the CIA mice.

The spleen index is associated with immunological functions. As shown in **Figure 3**, the spleen index of the CIA group increased compared with that of the normal control group. The treatment with various concentrations of AC (20, 30 and 40 mg/kg) resulted in significant decreases of the spleen index.

### AC Treatment Decreased Immune-Mediated Inflammation and Joint Damage in CIA Mice

**FIGURE 4:**
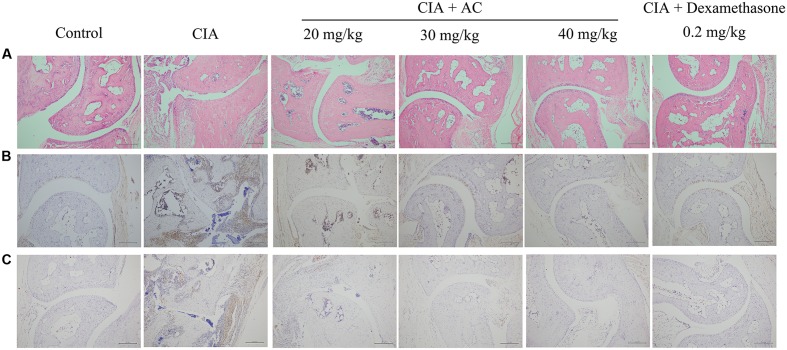
**Progression of LPS-enhanced arthritis was blocked by treatment with AC.** Male DBA/1J mice were immunized with CII, and LPS was administered on day 28 to induce LPS-enhanced CIA as described in “Materials and Methods.” **(A)** Histological evaluations of anti-arthritis effects of AC were performed using joint slides stained with H&E (original magnification ×100). **(B)** TNF-α expression in the joint on day 51 was evaluated by immunohistochemical staining (original magnification ×100). **(C)** Immunohistochemical analysis of CD68 was performed on sections of the ankle joint from healthy mice and CIA mice treated with vehicle or AC (original magnification ×100).

Upon LPS treatment, severe cartilage and bone erosions, accompanied by large amounts of inflammatory cell infiltration and pannus formation, were observed in the sections obtained from the CIA mice (**Figure 4**). In contrast, treatment with AC suppressed these changes, and the joint space was almost completely preserved in the AC-treated mice (**Figure 4A**). Development of arthritis in the CIA mice was associated with the increased numbers of macrophages and overproduction of TNF-α compared to these parameters in the normal control mice. AC significantly suppressed the TNF-α production in joint tissues as revealed by immunohistochemical staining (**Figure 4B**). The effective treatment consistently correlated with the reduction of CD68-positive macrophages. To further explain the anti-arthritis effects of AC, we investigated the macrophage population changes in joint tissue. As shown in **Figure 4C**, compared with the normal control mice, the number of CD68-positive cells increased in the synovium of the CIA group, while significantly decreased in that of the AC-treated group.

### AC Does Not Affect Growth of RAW 264.7 Cells

**FIGURE 5:**
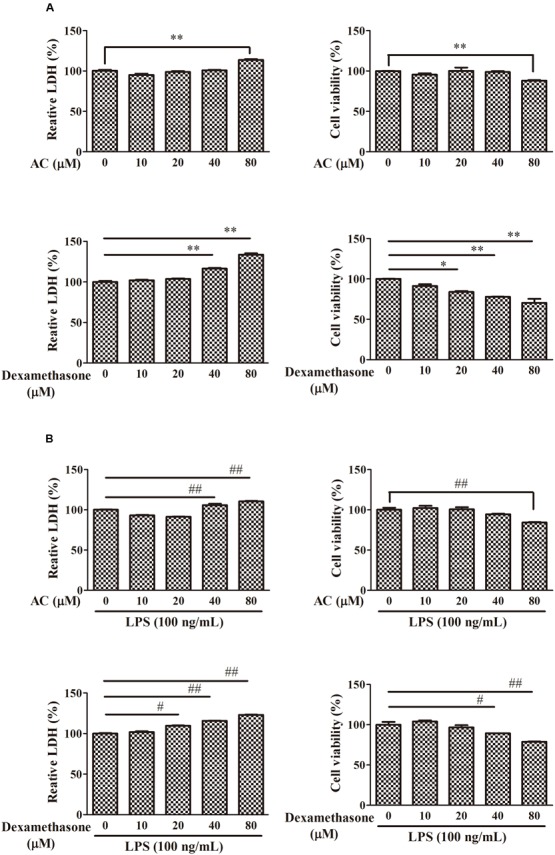
**Effects of AC and dexamethasone on the growth of RAW 264.7 cells.** **(A)** Cells were incubated in a complete medium with the indicated concentrations of AC and dexamethasone for 24 h, and cell viability was measured by the LDH and MTT assay. **(B)** Macrophages were treated with AC and dexamethasone (10, 20, 40, and 80 μM) and exposed to LPS (100 ng/mL) for 24 h. Cellular viability was measured using the LDH and MTT assay. Each value represents the mean ± SD of three independent experiments. ^∗^*P* < 0.05, ^∗∗^*P* < 0.01 compared with the control group; ^#^*P* < 0.05, ^##^*P* < 0.01 compared with the LPS-treated group.

To examine the cytotoxicity of AC, the LDH and MTT assay were used to evaluate the growth of RAW 264.7 cells. As shown in **Figure 5**, treatment with 10, 20, or 40 μM AC for 24 h had no significant effect on the cell viability; however, 80 μM AC decreased the cell viability by 20%. While dexamethasone exhibited significant inhibitory effect on cell viability at concentrations of above 20 μM. Based on these data, the three concentrations of AC (10, 20, and 40 μM) that failed to be cytotoxic to RAW 264.7 cells were used in subsequent experiments.

### Effects of AC on LPS-Induced Expression of iNOS and COX-2 in RAW 264.7 Cells

**FIGURE 6:**
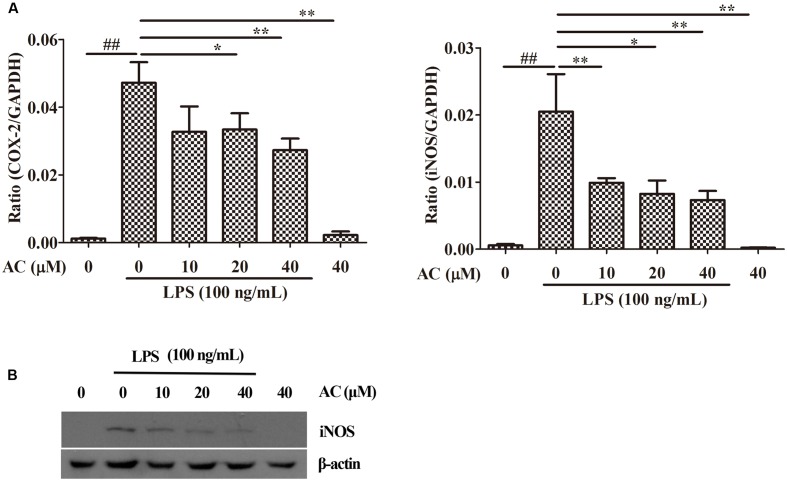
**Effects of AC on iNOS and COX-2 in RAW 264.7 cells.** **(A)** Cells were pretreated with AC (10, 20, and 40 μM) for 2 h and stimulated with LPS (100 ng/mL) for 3 h. The mRNA expression levels of iNOS and COX-2 were measured by real-time PCR. **(B)** Western blot analysis of the expression level of iNOS. Data were obtained from three independent experiments and are expressed as the mean ± SD. ^#^*P* < 0.05, ^##^*P* < 0.01 for the LPS-treated group vs. the normal control group; ^∗^*P* < 0.05, ^∗∗^*P* < 0.01 for the treatment groups vs. the LPS-treated group.

Nuclear factor-kappaB has been shown to mediate the production of the pro-inflammatory mediators iNOS and COX-2 by synoviocytes (Kunanusornchai et al., 2016; Wu et al., 2016b; Yang et al., 2016). Based on these data, we examined the effects of AC on the mRNA expression levels of iNOS and COX-2. As shown in **Figure 6A**, AC significantly inhibited the COX-2 and iNOS mRNA expression in a dose-dependent manner. Further, it was found that AC significantly inhibited the protein expression level of iNOS in a dose-dependent manner (**Figure 6B**).

### Effects of AC on mRNA Expression and Secretion Levels of TNF-α, IL-1β, and IL-6 in RAW 264.7 Cells

**FIGURE 7:**
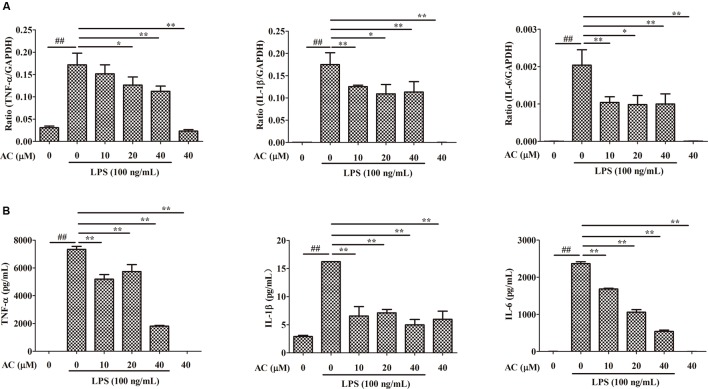
**Inhibitory effects of AC on** **(A)** mRNA expression and **(B)** secretion of TNF-α, IL-1β, and IL-6 in LPS-induced RAW 264.7 cells. Data were obtained from three independent experiments and are expressed as the mean ± SD. ^#^*P* < 0.05, ^##^*P* < 0.01 for the LPS-treated group vs. the normal control group; ^∗^*P* < 0.05, ^∗∗^*P* < 0.01 for the treatment groups vs. the LPS-treated group.

It is well known that pro-inflammatory cytokines play crucial roles in the pathogenesis of RA. To examine the anti-inflammatory effect of AC, we evaluated the mRNA expression levels of cytokines produced in LPS-induced RAW 264.7 cells. As shown in **Figure 7A**, the mRNA expression levels of TNF-α, IL-1β, and IL-6 significantly increased after LPS stimulation. Following AC treatment, the LPS-triggered TNF-α, IL-1β, and IL-6 expression was dose-dependently inhibited.

Enzyme-linked immunosorbent assays were used to evaluate the effect of AC on the secretion of pro-inflammatory cytokines by LPS-treated RAW 264.7 cells. Consistent with the changes in the mRNA expression levels, AC dose-dependently inhibited the secretion of TNF-α, IL-1β, and IL-6 (**Figure 7B**). In particular, the TNF-α production decreased to 1.81 ng/mL at an AC concentration of 40 μM compared to 7.34 ng/mL in LPS-treated RAW 264.7 cells. The results of this experiment were consistent with those of the animal experiments, in which AC significantly suppressed the TNF-α production in joint tissues.

### Effect of AC on Activation of Mitogen-Activated Protein Kinase Signaling Pathways in LPS-Stimulated RAW 264.7 Cells

**FIGURE 8:**
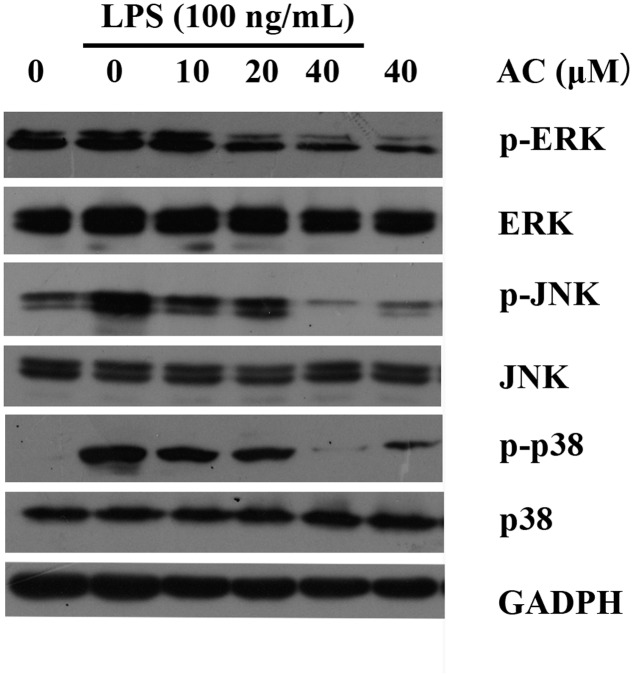
**Effects of AC on the activation of MAPK signaling pathways in LPS-stimulated RAW 264.7 cells.** RAW 264.7 cells were pretreated with the vehicle or the indicated concentrations of AC for 2 h before stimulation with LPS (100 ng/mL) for another 30 min. Western blot analysis was performed to detect the expression levels of p-ERK, ERK, p-JNK, JNK, p-p38 and p38.

It has been reported that stimulation of RAW 264.7 macrophages with LPS results in the activation of MAPKs, including ERK, p38, and JNK (Kumar et al., 2016). We next investigated whether AC modulated these signaling proteins in LPS-induced RAW 264.7 cells. As shown in **Figure 8** and Supplementary Figure S1, the results indicated that AC (10, 20, and 40 μM) dose-dependently attenuated the LPS-stimulated phosphorylation of ERK. Similarly, AC showed a strong inhibitory effect on the phosphorylation of JNK and p38 MAPK in response to LPS stimulation (**Figure 8**).

### Effects of AC on TLR4 and NF-κB Nuclear Translocation in LPS-Induced RAW 264.7 Cells

**FIGURE 9:**
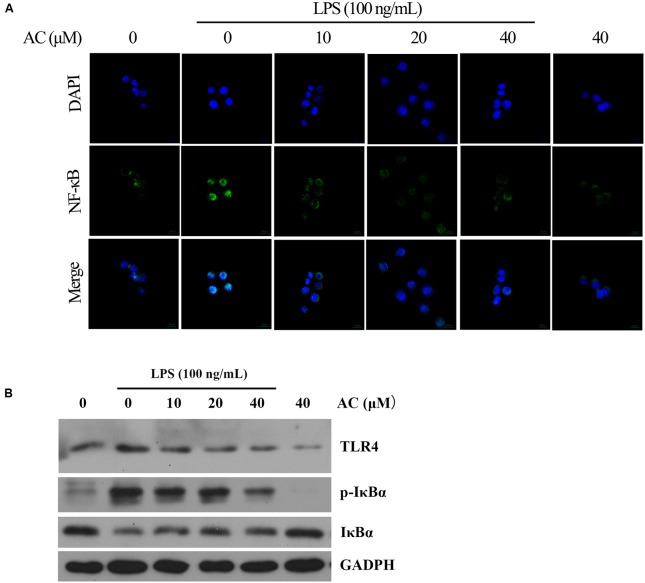
**(A)** Effects of AC on the nuclear NF-κB translocation in LPS-induced RAW 264.7 cells. RAW 264.7 cells were pretreated with AC (10, 20, and 40 μM) for 2 h, followed by incubation with LPS (100 ng/mL) for 30 min, and nuclear translocation of NF-κB was observed by confocal microscopy (×63). **(B)** Effects of AC on the expression levels of TLR4, the cytoplasmic IκBα as well as the phosphorylation level of cytoplasmic IκBα.

TLR4 signaling plays an important role in inflammatory response. Activation of TLR4 by LPS induces NF-κB and MAPKs activation in RAW264.7 cells exposed to LPS (Huang and Pope, 2009; Wu et al., 2016a). Therefore, the expression level of TLR4 was detected by western blot analysis. The result showed that AC significantly decreased the expression of TLR4 in a dose-dependent manner (**Figure 9B**).

Nuclear factor-kappaB is a key transcriptional regulator in immune and inflammatory responses, and it regulates the production of several cytokines, including TNF-α, IL-1β, and IL-6 (Mu et al., 2016). Therefore, we hypothesized that the anti-inflammatory effects of AC might be associated with the NF-κB pathway. To confirm this hypothesis, we used immunofluorescence staining to determine the NF-κB activity. As shown in **Figure 9A**, the immunofluorescence images revealed that the basal NF-κB p65 was distributed in the cytoplasm of RAW 264.7 cells, while nuclear translocation of p65 increased after LPS stimulation. In contrast, this translocation was dose-dependently inhibited by AC treatment, and no obvious change in the NF-κB activity was observed in the presence of AC (40 μM) alone. NF-κB translocation into the nucleus is preceded by the phosphorylation, ubiquitination, and proteolytic degradation of IκBα (Torices et al., 2015). To determine whether the inhibitory action of AC on LPS-induced NF-κB activation was due to its effect on IκBα degradation, we determined the expression and phosphorylation levels of cytoplasmic IκBα by the western blot assay. The results showed that the LPS-induced activation of IκBα was significantly inhibited by AC treatment (**Figure 9B**). In addition, a significant decrease of the expression level of IκBα was observed in LPS-induced RAW 264.7 cells, while it increased dose-dependently in the presence of AC.

## Discussion

Macrophages are found in large numbers throughout the synovium of RA patients. Macrophage-derived cells, named type A synoviocytes, constitute part of the healthy synovial lining; however, numerous activated macrophages of other subtypes are accumulated in the synovial inflammatory infiltrate and pannus-cartilage interface. Activated macrophages produce many pro-inflammatory mediators, including TNF-α, IL-6, and IL-8, as well as tissue-degrading enzymes, which contribute to the inflammatory response and bone destruction. Furthermore, the number of synovial macrophages, as indicated by the expression of CD68, has been found to correlate with joint erosion in RA (Udalova et al., 2016; Yeo et al., 2016). In this study, the number of CD68-positive macrophages significantly increased in synovial tissues of CIA mice and strongly decreased in AC-treated CIA mice in a dose-dependent manner (**Figure 4**).

Nitric oxide is synthesized by the iNOS enzyme, and iNOS and COX-2 are often co-expressed in inflammatory tissues (**Figure 6B**). Excessive production of NO and COX-2 can lead to a simultaneous release of prostaglandin E2 (PGE2) in degenerative and inflammatory arthritis. In our previous study, AFS was found to show anti-arthritic effects on type II CIA in rats. We have determined that AC significantly inhibited the NO production by LPS-stimulated macrophage cell lines as well. Among the main active constituents of AFS, AC showed the strongest anti-inflammatory activity (Huang et al., 2014). Thus we thought AC was superior to the other compounds and examined anti-rheumatic effects of AC in a mouse model in the present study. It could be deduced from the structures of all these six tested compounds that they might act through the same target, since they are all triterpenoid saponins with similar substituent glycosyl group(s). The difference in anti-inflammatory activity might be due to the steric hindrance of glycosyl group(s) and the numbers of H-bond formed between the glycosyl group(s) and its target. It would be worthy that although the dose of dexamethasone used in the present study was lower than AC, the toxicity of dexamethasone was significantly greater than AC *in vitro* and *in vivo*. It was observed in this study that the weight of dexamethasone-treated CIA mice was significantly reduced, while that of AC-treated group had no obvious change (**Figure 2**), suggesting the application of AC as a novel therapeutic agent for human RA or as an effective lead compound for the discovery of anti-RA drugs.

Previous reports have suggested that agents simultaneously inhibiting the NO and PGE2 production may show synergistic effects against chronic inflammation (Sakaguchi et al., 2006; Li et al., 2013). The results of our study revealed that AC at various concentrations downregulated the COX-2 and iNOS enzymes at the mRNA expression level and iNOS at the protein expression level in LPS-stimulated RAW 264.7 cells. Although the exact pathology of RA is unclear, proinflammatory cytokines and mediators are involved in the pathogenesis of arthritis (McInnes and Schett, 2007; Klareskog et al., 2009). Published data have revealed that TNF-α and IL-1β could promote the RA progress through mediators such as COX-2 and MMPs (Wu et al., 2016b). Among cytokines, TNF-α plays a vital role in RA, inducing inflammatory cytokine cascades *in vivo* and facilitating the osteoclast differentiation, formation, activation, and apoptosis, which ultimately leads to the erosion of the articular cartilage and bone (Yuan et al., 2012). A previous study has reported that human TNF-transgenic mice are an animal model of spontaneous development of arthritis to study therapeutics for RA. A TNF-α-specific antibody and a soluble TNF receptor showed a prophylactic efficacy in the mouse model (Bevaart et al., 2010; Wang et al., 2016). In RA patients, the joint fluid and plasma contain high concentrations of IL-6, which is critical for the differentiation and activation of osteoclasts and bone resorption and enhances the vascular permeability of synovial tissue through stimulation of excess production of vascular endothelial growth factor (Yao et al., 2014). Furthermore, numerous studies have reported that IL-6 blocks the regulatory T (Treg) cell activity and inhibits Treg generation induced by transforming growth factor beta. Moreover, IL-6 promotes T helper 17 (Th17) cell differentiation as a regulator of the Treg/Th17 cell balance (Assier et al., 2010; Yoshida and Tanaka, 2014). Our results demonstrated that AC significantly suppressed the LPS-induced mRNA expression and secretion of TNF-α, IL-1β, and IL-6 *in vitro*, and the TNF-α tissue production extremely decreased in AC-treated CIA mice (**Figure 7**). Hence, AC could exert its anti-arthritic effects, at least partially, through inhibition of excessive production of iNOS, COX-2, TNF-α, IL-1β, and IL-6.

Mitogen-activated protein kinase pathways are critical in regulating progressive joint destruction in RA. The MAPK family consists of three main subfamily members, ERK, p38, and JNK. JNK and p38 MAPKs play critical roles in regulating synovial invasion, inflammatory cytokine secretion by activated macrophages, and collagenase synthesis by RA FLSs, while ERK MAPK promotes the production of several cytokines and pannus formation (Ganesan et al., 2016; Ross et al., 2016; Yang et al., 2016). Thus, it has been suggested that inhibitors targeting the p38 and JNK MAPK pathways have anti-inflammatory activity (Kaminska, 2005). In the present study, we detected inhibitory effects of AC on the LPS-induced phosphorylation of MAPKs in RAW 264.7 cells. In particular, we found that AC inhibited the LPS-triggered phosphorylation of p38 and JNK but had little effect on that of ERK. These results suggest that the effects of AC on the production of inflammatory mediators and cytokines are likely mediated through blocking of the p38 and JNK signaling pathways in monocytic cells (**Figure 8**).

It is well established that NF-κB signaling plays a vital role in the progress and development of RA by regulating the transcription of pro-inflammatory mediators such as iNOS, COX-2, TNF-α, IL-1β, and IL-6 in activated macrophages (Li et al., 2013; Yang et al., 2016). The activation of NF-κB and translocation of the p65 subunit to the nucleus is preceded by the phosphorylation, ubiquitination, and degradation of IκBα (Torices et al., 2015). In this study, we showed that AC significantly attenuated the LPS-induced IκB phosphorylation and degradation as well as the nuclear translocation of p65 in LPS-stimulated cultured RAW 264.7 cells, as demonstrated by the results of western blot and immunocytochemistry for NF-κB p65 (**Figure 9**). LPS from the cellular wall of Gram-negative bacteria plays a key role in producing an inflammatory response. Many studies have reported that LPS triggers the immune response via TLR4 and activates the NF-κB and MAPKs signaling pathways to regulate the production of inflammatory cytokines, chemokines and tissue destructive enzymes (Park et al., 2013). To investigate the upstream signaling transduction mechanism, we determined the effect of AC on the expression level of TLR4 in LPS-induced RAW 264.7 cells. The results showed that AC decreased the expression level of TLR4 in a dose-dependent manner (**Figure 9B**), suggesting that AC might suppressed the activation of NF-κB and MAPK signaling pathways by the down-regulation of TLR4. In addition, it has been reported that triterpenoids exhibited potential anti-inflammatory activity by targeting PDE4 (Tan et al., 2017), Keap1-Nrf2 (Zhuang et al., 2014), COX-1 and/or COX-2 (Fu et al., 2010; Niu et al., 2014; Rauf et al., 2016). Whether AC could inhibit the function of the above proteins are worthy of further investigation.

In summary, our study provides evidence that AC causes a therapeutic effect on an LPS-enhanced CIA mouse model through inhibition of production of proinflammatory cytokines such as TNF-α, IL-1β, and IL-6 as well as other mediators such as iNOS and COX-2. Furthermore, AC down-regulates TLR4, deregulates the activities of MAPK signaling pathways and suppresses the NF-κB signaling pathway. The findings from the present *in vitro* and *in vivo* study suggest that AC has the potential to be developed as a novel therapeutic agent for human RA.

## Author Contributions

Participated in research design: ZL, M-ML, and W-CY. Conducted experiments: QL, X-HX, L-BH, H-YJ, ZL, and M-ML. Contributed new reagents or analytic tools: QL, X-HX, ZL, and M-ML. Performed data analysis: QL, X-HX, L-BH, ZL, M-ML, H-YJ, and YW. Wrote or contributed to the writing of the manuscript: QL, X-HX, ZL, M-ML, and W-CY.

## Conflict of Interest Statement

The authors declare that the research was conducted in the absence of any commercial or financial relationships that could be construed as a potential conflict of interest.

## Abbreviations

AC: anhuienoside C
AFS: *Anemone flaccida* crude triterpenoid saponins
BCA: bicinchoninic acid
CD68: differentiation 68
CFA: complete Freund’s adjuvant
CIA: collagen-induced arthritis
CII: type II collagen
COX-2: cyclooxygenase 2
DAPI: 4′,6-diamidino-2-phenylindole
DMEM: Dulbecco’s Modified Eagle’s Medium
DMSO: dimethyl sulfoxide
ECL: chemiluminescence
ELISA: enzyme-linked immunosorbent assay
ERK: extracellular signal-regulated kinase
FBS: fetal bovine serum
FLSs: fibroblast-like synoviocytes
GAPDH: glyceraldehyde 3-phosphate dehydrogenase
H&E: hematoxylin and eosin
IFA: incomplete Freund’s adjuvant
IL-β: interleukin-1β
IL-6: interleukin-6
iNOS: nitric oxide synthase
IκBα: inhibitor of kappa B
JNK: Jun N-terminal kinase
LDH: lactate dehydrogenase
LPS: lipopolysaccharide
MAPK: mitogen-activated protein kinase
MMPs: matrix metalloproteinases
MTT: 3-[4,5-dimethylthiazol-2-yl]-2,5-diphenyltetrazolium bromide
NF-κB: nuclear factor-kappaB
NMR: nuclear magnetic resonance
NO: nitric oxide
NSAIDs: nonsteroidal anti-inflammatory drugs
PBS: phosphate-buffered saline
PVDF: polyvinylidene fluoride
RA: rheumatoid arthritis
SD: standard deviation
SDS-PAGE: sodium dodecyl sulfate-polyacrylamide gel electrophoresis
SPF: specific pathogen-free
TLR4: toll-like receptor 4
TNF-α: tumor necrosis factor
