# Supplementary figures and images for: Anhuienoside C Ameliorates Collagen-Induced Arthritis through Inhibition of MAPK and NF-κB Signaling Pathways
Source: Front Pharmacol. 2017 May 26;8:299. doi: 10.3389/fphar.2017.00299 (PMC5445104; doi:10.3389/fphar.2017.00299)

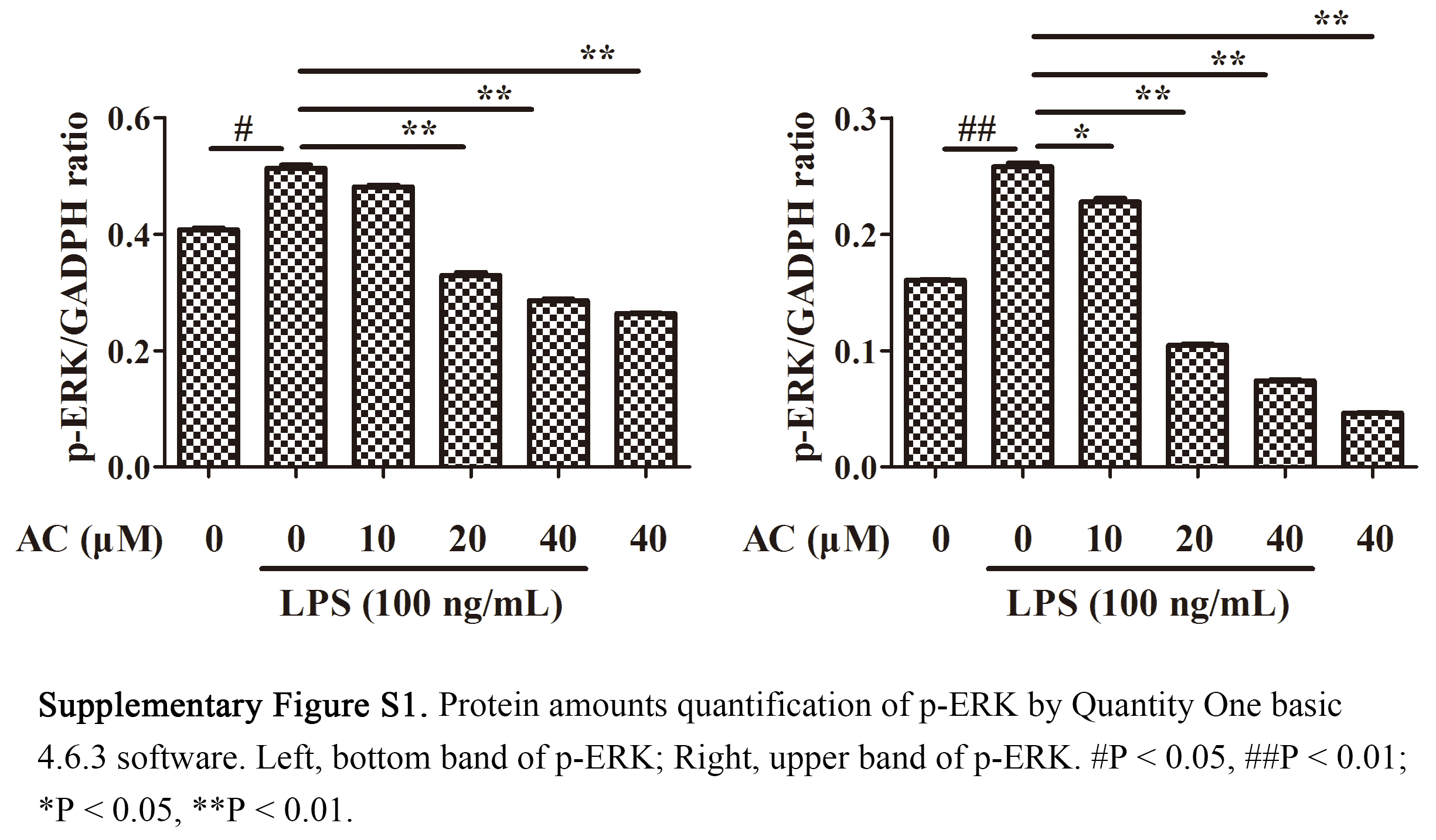

Supplement: Supplementary file 1 [file Image_1.TIF]
